# Cytogenomic profiling of breast cancer brain metastases reveals potential for repurposing targeted therapeutics

**DOI:** 10.18632/oncotarget.3786

**Published:** 2015-04-24

**Authors:** Aliccia Bollig-Fischer, Sharon K. Michelhaugh, Priyanga Wijesinghe, Greg Dyson, Adele Kruger, Nallasivam Palanisamy, Lydia Choi, Baraa Alosh, Rouba Ali-Fehmi, Sandeep Mittal

**Affiliations:** ^1^ Department of Oncology at Wayne State University, Detroit, Michigan, USA; ^2^ Department of Neurosurgery at Wayne State University, Detroit, Michigan, USA; ^3^ Department of Obstetrics/Gynecology at Wayne State University, Detroit, Michigan, USA; ^4^ Department of Pathology at Wayne State University, Detroit, Michigan, USA; ^5^ Karmanos Cancer Institute, Detroit, Michigan, USA; ^6^ Henry Ford Health System, Detroit, Michigan, USA

**Keywords:** breast cancer, brain metastases, copy number variation, oncogenes, targeted therapy

## Abstract

Breast cancer brain metastases remain a significant clinical problem. Chemotherapy is ineffective and a lack of treatment options result in poor patient outcomes. Targeted therapeutics have proven to be highly effective in primary breast cancer, but lack of molecular genomic characterization of metastatic brain tumors is hindering the development of new treatment regimens. Here we contribute to fill this void by reporting on gene copy number variation (CNV) in 10 breast cancer metastatic brain tumors, assayed by array comparative genomic hybridization (aCGH). Results were compared to a list of cancer genes verified by others to influence cancer. Cancer gene aberrations were identified in all specimens and pathway-level analysis was applied to aggregate data, which identified stem cell pluripotency pathway enrichment and highlighted recurring, significant amplification of *SOX2*, *PIK3CA*, *NTRK1*, *GNAS*, *CTNNB1*, and *FGFR1*. For a subset of the metastatic brain tumor samples (n=4) we compared patient-matched primary breast cancer specimens. The results of our CGH analysis and validation by alternative methods indicate that oncogenic signals driving growth of metastatic tumors exist in the original cancer. This report contributes support for more rapid development of new treatments of metastatic brain tumors, the use of genomic-based diagnostic tools and repurposed drug treatments.

## INTRODUCTION

Despite advances in the treatments for breast cancer, effective treatments for brain metastases remain elusive. It is estimated that 30% of breast cancer patients will develop a brain metastasis, and with extended patient survival times due to improved drug management this rate is expected to increase [1, 2]. There are currently no biomarkers or tests to predict which patients will be afflicted and typically a brain tumor is only discovered when a patient exhibits debilitating neurological symptoms. Patients with metastatic brain tumors do not respond to chemotherapy leaving only surgical resection and radiotherapy as treatment options with an average survival of only 6-9 months. If left untreated, the median survival for a patient is 1 month.

The brain presents a unique and complex tissue microenvironment and the colonization and formation of metastatic tumors depends on interactions of the properties of the microenvironment and the phenotype of the colonizing metastatic breast cancer cells [3, 4]. This concept must now be reconciled with knowledge that cancer is ultimately a genetic disease with pathobiology driven by somatic gene mutations, i.e., gain-of-function mutations in oncogenes and loss-of-function mutations in tumor suppressors. Yet an examination of the biomedical literature reveals a relative lack of molecular genomic characterization of brain metastases [5], which could lead to effective therapeutic interventions and better patient outcomes as evidenced by advancements in therapies guided by molecular targets to treat primary breast tumors, e.g., drugs targeting estrogen receptor-alpha (ERα) or the epidermal growth factor receptor ERBB2/HER2 [6]. The projected feasibility of molecularly-targeted therapies for metastatic brain tumors is supported by a growing body of empirical evidence that small tyrosine kinase inhibitors and antigen-targeted drugs can traverse the blood-brain barrier and result in clinical benefit for patients harboring metastatic brain lesions [7–12].

By analyzing copy number aberrations in surgically-resected metastatic brain tumors and matched primary breast tumor specimens, the present work provides insights as to the nature of the genomic lesions that are likely molecular contributors to brain metastases. Furthermore, our bioinformatics analysis highlights various oncogene amplification events that are the focus of molecularly targeted FDA-approved drugs and drugs in clinical trials that might be redirected to treat metastatic brain tumors thus providing a shorter timeline for the design of new treatment strategies over the development of novel therapeutic agents.

## RESULTS

### Patient population

As shown in Table 1, all 10 study patients were female with an average age at primary breast cancer diagnosis of 51±11 years. The average interval to the diagnosis of brain metastasis was 3.5±2 years. Out of the 10 patients, 5 were hormone receptor positive (with 3 of those also ERBB2/HER2 positive), 4 were triple negative, and the hormone receptor profile was unknown in 1 patient.

**Table 1:** Study population demographics, breast tumor profiles and systemic treatments

Subject ID	Age at Diagnosis (years)	Interval to Brain Metastasis (years)	Primary Tumor Receptor Profile	Primary Tumor Histologic Subtype	Systemic Treatment for Primary Tumor
09–34	38	6.5	ER+PR+H2N-	HR+	neoadjuvant AC × 4 cycles, no docetaxel, little response to chemotherapy, proceeded to MRM with T4N2 disease
09–35	50	5	n/a	n/a	chemotherapy (unspecified) and breast RT after with primary disease
12–16	65	3	ER/PR+	HR+	AC+T, post-mastectomy chest wall RT, anastrozole
12–23	41	1	ER-PR-H2N-	TNBC	No chemotherapy because of multiple medical comorbidities (ESRD, DM)
12–25	58	2.5	ER+PR-H2N+	HR+ HER2+	chemotherapy (unspecified), whole breast RT after lumpectomy
12–33	39	5	ER+PR+H2N+	HR+ HER2+	AC × 6 cycles, TH, anastrozole, and zoledronic acid
12–37	41	6	ER-PR-H2N-	TNBC	5-FU, epirubicin and cyclophosphamide × 6 cycles, whole breast RT then MRM 2 months later followed by post-mastectomy RT, then incisional recurrence treated with RT and capecitabine, then lung metastasis treated with surgery and docetaxel, then gemcitabine, then paclitaxel (each drug for disease progression while on previous chemotherapy agent)
13–02	62	2	ER-PR-H2N-	TNBC	AC+T 2011, post-mastectomy RT
13–03	60	1.5	ER-PR-H2N-	TNBC	neoadjuvant AC+T, whole breast RT after lumpectomy
13–19	58	2.5	ER-PR+H2N+	HR+ HER2+	ACTH, whole breast RT after lumpectomy

***Abbreviations:*** n/a: not available; ER: estrogen receptor; PR: progesterone receptor; H2N: HER-2/neu; TNBC: triple negative breast cancer; AC: doxorubicin and cyclophosphamide; AC+T: doxorubicin, cyclophosphamide, and docetaxel; 5-FU: fluorouracil; MRM: modified-radical mastectomy; RT: radiation therapy; ESRD: end-stage renal disease; DM: diabetes mellitus; TH: docetaxel and trastuzumab (Herceptin); ACTH: doxorubicin and cyclophosphamide followed by paclitaxel and trastuzumab

### Re-occurring cancer gene amplifications in breast metastatic brain tumors

**Figure 1:**
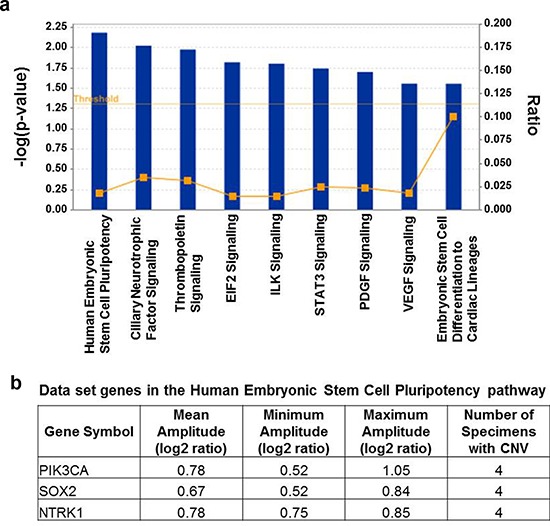
Result of pathways analysis of the set of genes showing repeated copy number gains in DNA from metastatic brain tumor tissue **a.** Significant over-representation (*p* ≤ 0.05) is observed for each of the displayed canonical pathways and the most over-represented was the *Stem Cell Pluripotency* pathway (*p* < 0.01). Ratio on the right vertical axis refers to the number of genes from the analyzed data set mapping to each pathway divided by the total number of genes in the pathway. **b.** The genes mapping to the Stem Cell Pluripotency pathway from the analyzed data set that showed CNV ≥0.4 log2 ratio in ≥40% of the breast metastatic brain tumors included in the study. The complete list of 55 genes meeting these thresholds is in Supplementary Table 2.

Macro-colonization is described as the rate-limiting step in metastatic tumor formation, accounting for fewer than 0.1% of cancer cells that enter the circulatory system [13–16]. This suggests that the spectrum of mutation-activated oncogenes driving the processes of colonization and/or metastatic tumor outgrowth is limited, and that causative lesions would be recurrent in patient specimens. We investigated this using the copy number aberration data from our aCGH analysis of 10 metastatic brain tumors and identified recurring amplifications in 55 cancer-linked genes based on the minimum condition that gene amplification occurred in 40% of specimens at a log2 ratio greater or equal to +/−0.4 (Supplementary Table 2). According to these criteria there were no recurring gene losses. Among the recurrent gene amplifications were oncogenes *PIK3CA* and *MYC*, also known to be frequently amplified in breast cancers. Ingenuity Pathways Analysis (IPA) of the 55 gene set showed over-representation for canonical pathways categorized under Cellular Growth, Proliferation and Development descriptors; and the most over-represented was the *Stem Cell Pluripotency* pathway (*p<0.01*; Figure 1); genes in this pathway showing copy number aberrations in the metastatic tumors were *PIK3CA*, *SOX2* and the neurotrophin receptor *NTRK1*.

### Cancer gene lesions with highest amplitude and potential for therapy

**Figure 2:**
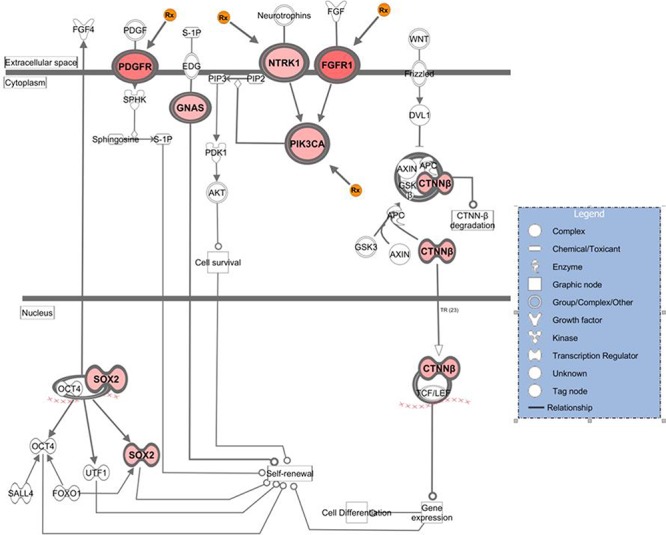
Genes that showed high-level amplification in the breast metastatic brain tumors that map to the stem cell pluripotency pathway The figure is the output of Ingenuity Systems pathway analysis (IPA) tool. Genes included in the analysis demonstrated ≥0.8 log2 ratio in at least one specimen of 10. Intensity of color reflects relative amplitude of copy number gain detected, where darkest red indicates highest amplitude (the copy number log2 ratio data for genes here are in Supplementary Table 3). The orange Rx symbol points to those genes where targeted inhibitory drugs are available.

We then identified genes showing the highest levels of amplification in patient tumor specimens with the rationale that patients receiving the greatest benefit from molecularly targeted drugs have tumors with high target levels. We set the minimum amplitude threshold at a log2 ratio of +/−0.8 and producing a list of 109 genes (Supplementary Table 3). It was subjected to IPA and again the *Stem Cell Pluripotency* pathway rose to significance. By focusing on genes showing higher amplitude copy number aberrations as opposed to those that were recurrent, the numbers of analyzed genes mapping to this pathway increased, to include *SOX2*, *PIK3CA*, *NTRK1* as well as *GNAS*, *CTNNB1*, and *FGFR1* (Figure 2). In addition, among all the genes in this analyzed data set 13 amplified genes were identified as potential drug targets, or clinically informative biomarkers (Table 2).

**Table 2:** Copy number variations detected in breast metastatic brain tumors for cancer genes that are the target of existing drugs

Gene	Number Samples Identified	Amplification Range(log2 ratio)	Gene Function	Inhibitor Drug Examples*
CCND1	1	2.11	other	daunorubicin, gemtuzumab
DDR2	1	0.85	kinase	regorafenib
ERBB2	2	1.83-2.54	kinase	trastuzumab, lapatinib, erlotinib
FGFR1	1	1.75	kinase	sorafenib, dexamethasone
JAK2	1	2.17	kinase	ruxolitinib
JAK3	1	0.81	kinase	tofacitinib, R-348
KDR	1	0.88	kinase	cabozantinib, bevacizumab
KIT	2	0.86-0.88	transmembrane receptor	dasatinib, sunitinib
MUC1	2	0.85-0.92	transcription regulator	HuHMFG1
NTRK1	1	0.85	kinase	regorafenib
PDGFRA	2	0.88-2.26	kinase	sunitinib, pazopanib, imatinib
PIK3CA	2	0.84-1.05	kinase	SF1126, PX-866
RAF1	1	0.86	kinase	vemurafenib, sorafenib

* Not a complete list.

### Comparing brain metastases with matched primary cancer tissue

**Figure 3:**
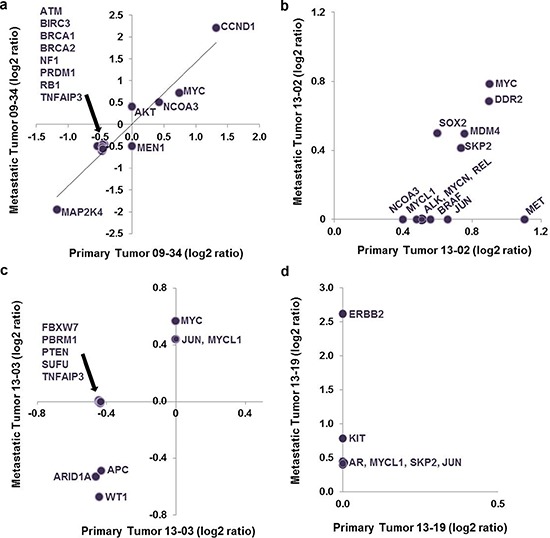
Plot of log2 ratio data for cancer gene aberrations discovered in matched primary and metastatic tumor specimens Panels **a–d.** show the individual results of analysis of matched primary breast and metastatic cancer specimens from four patients. Representing one side of the possible outcomes spectrum, a. displays outcomes where the log2 ratios for cancer gene aberrations were identical in the primary and metastatic tumors. d. shows the extreme example where no lesions were discovered in the primary tumor, but 6 cancer gene aberrations were observed in the metastatic tumor with *ERBB2* having the highest copy number gain.

The relationship between cancer driver genes in primary breast cancer and those in metastatic brain tumors remains incompletely understood. Here, we pose the hypothesis that brain colonizing lesions pre-exist in the primary tumor specimen. It is an attractive hypothesis because, if true, this knowledge could allow development of lab tests to predict the propensity toward a metastatic event in the brain, or suggest therapeutic interventions to thwart the development of impending metastatic brain tumors. On the other hand, others put forth the possibility that important genomic lesions develop beyond the primary tumor or at the site of metastases [17]. Considering both concepts, we undertook a descriptive comparison of gene lesions for 4 sets of matched primary and metastatic specimens. Because only FFPE primary breast tumor specimens were available, we analyzed FFPE and fresh frozen metastatic brain tumor tissue samples and discovered the same cancer gene CNV results for the same patient specimens exposed to different preparation and storage. Graphs in Figure 3 display gene symbols and associated aberrant copy number (log2 ratio) identified in each FFPE metastatic or primary specimen; the graphs show if a particular gene lesion was identified in both the primary and metastatic tumor or if it was unique to either tumor in each set. Thus evidence from analysis of cancer gene copy number aberrations indicates that at a gene lesion level metastatic brain tumors can be highly similar or divergent from the cancer of origin.

**Figure 4:**
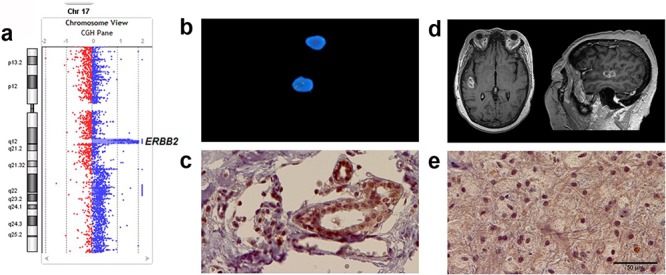
Various clinically viable approaches to measure ERBB2 in metastatic and primary tumors from a single patient both verify results and reveal complexities in interpreting outcomes In this specific patient example, **a.** according to aCGH analysis high copy number gain for *ERBB2* was observed in the metastatic brain tumor specimen. *ERBB2* is the only translated gene that mapped to the focal amplification coordinates HG19: Chr17: 37863329-37866691. **b.** The result of FISH analysis of the original biopsied breast cancer specimen concluded that there was normal diploid copy number for *ERBB2* in DNA, and this agrees with results of our aCGH analysis of the primary specimen that were also negative for *ERBB2* amplification. **c.** IHC analysis of the primary breast cancer specimen indicates that although not DNA amplified, ERBB2 is upregulated at the protein level in the primary specimen. **d.** MRI scan of patient (13-19) with right temporal brain metastasis from primary breast cancer. **e.** IHC of the metastatic brain lesion shows relatively homogenous immunostaining for ERBB2. Scale bar is 50 μM and also applies to panel c.

Another important facet to our study of these paired specimens emerged that underscores the complexities of measuring oncogene mutations in tumor samples. Figure 4 provides an illustrative case. A graph of data from aCGH analysis of the metastatic tumor labeled 13-19 shows a high-level, focused amplification encompassing the entire *ERBB2* gene on chromosome 17q12 (Figure 4a). Analysis of the matched primary tumor by aCGH showed that specimen was negative for *ERBB2* amplification. This negative result concurs with the clinical pathology report for the original breast cancer biopsy specimen, where *ERBB2* gene was not amplified according to FISH (Figure 4b). However, the original pathology report also describes the primary breast cancer specimen as being ERBB2 positive (HER2 positive score 3+) according to IHC testing, which we also demonstrated by repeating ERBB2 protein-level staining of the primary specimen (Figure 4c). In addition, IHC analysis of the 13-19 metastatic brain tumor specimen showed strong ERBB2 staining (Figure 4d).

## DISCUSSION

Breast cancer patients who suffer metastatic brain tumors are in dire need of improved treatment options. There is mounting evidence that metastatic cancer patients will one day benefit from informed molecularly-targeted therapies based on each individual's clinical and oncologic profile. There is growing literature on the molecular underpinnings of breast cancer metastatic brain tumor growth [5, 17–27], but even collectively from the literature the numbers of specimens reported on remain limited; also for many reports the number of features analyzed were limited. As such, what remains to be understood is the topic of this report: what are the mutated oncogenes or tumor suppressors found in breast metastatic brain tumor specimens? Our analyses describe 10 individual metastatic specimens and a subset of 4 available matched primary cancer specimens to appreciate the gene copy number lesions that are found in the metastatic tumors; honing in those that may present therapeutic options or important insights to tumor development.

The amplified oncogenes identified in metastatic tumor specimens included genes known to be frequently amplified in breast cancers such as *PIK3CA* and *MYC*. Pathway analysis of gene sets comprising amplifications showed significant enrichment for the Stem Cell Pluripotency pathway, highlighting our discovery of recurring significant amplification of *SOX2*, *PIK3CA*, *NTRK1*, *GNAS*, *CTNNB1*, and *FGFR1* in the samples analyzed. This contributes genomic evidence to reinforce the importance of cancer stem-like cells in promoting metastatic brain tumors. Cancer stem-like cells represent a minor subset of cells within a tumor and similarly to stem cells, they maintain the capability for unlimited self-renewal and are a potential cell type from the primary tumor that is motile and invasive and initiates a metastatic lesion [28–30].

Copy number gains are activating for oncogenes such as *ERBB2* (identified in 2 of 10 metastatic tumor specimens analyzed) because they cause corresponding aberrant overexpression of gene transcripts. Among the more novel genes we uncovered in our analysis (amplified in 4 of 10 metastatic tumors analyzed) was the neurotrophin receptor *NTRK1*, a tyrosine kinase growth factor receptor. According to database queries *NTRK1* is normally highly expressed in central and peripheral nervous system tissues and only the lowest levels of expression is detected in benign breast tissues [31, 32]. Also of interest to the outcomes of our study, according to data from The Cancer Genome Atlas (TCGA) — accessed and analyzed via the cBioPortal — *NTRK1* copy number gains occur in 12% of invasive breast carcinomas (of 962 samples) [33, 34]. Altogether, the data provide early evidence to indicate that amplification and/or overexpression of *NTRK1* has a potential role in the incidence of metastatic breast tumors and warrants further study.

The last topic of our discussion addresses whether or not primary breast cancers harbor the genomic lesions that are causal in the development of metastatic brain tumors; as opposed to a scenario where mutations evolve outside of the primary tumor, perhaps at the site of metastases prior to macro-colonization. The number of primary specimens available to us for this study was particularly limited because often patients with advanced or metastatic cancers come to the Karmanos Cancer Institute after having had their primary cancer diagnosed and treated elsewhere. However, based on our analyses of cancer gene CNV in 4 matched primary breast and metastatic brain tumors, we witnessed the full spectrum of what might be observed.

One primary and metastatic tumor set showed nearly identical cancer gene aberration profiles (Figure 3a). For this pair *CCND1* specifically demonstrated a high amplification in the primary cancer (1.32 log2 ratio amplification spanning 1.5Mb sequence and 13 genes); a similar amplified region containing CCND1 was more focused and of increased amplitude in the metastatic tumor (a 752,615 base pair, 2.2 log2 ratio amplification comprising only 6 genes, including *CCND1*, *MRGPRD*, *MRGPRF*, *TPCN2*, *MYEOV* and *ORAOV1*). *AKT2* amplification was detected only in the metastatic tumor samples, as part of a broad low level amplification event (0.4 log2 ratio, 2.5Mb comprising more than 75 genes/transcripts). Together, the data suggest that *AKT2* is not causal for the metastatic lesion, but that *CCND1* likely has a role in the primary breast cancer that is reinforced in the metastatic cancer.

Two paired primary-metastatic tumor sets demonstrated a set of conserved cancer gene aberrations, though one set showed additional cancer gene lesions in the primary cancer specimen and the other had gains in the metastasis. In one instance, for only the primary tumor (set 13-02 Figure 3b) *MET* was part of a focal amplification event (1.1 log2 ratio spanning 287,678 base pairs and 2 genes: *MET* and *CAV1*); also, broad amplification events comprising *MYC* and *DDR2* were identified in both primary and metastatic samples (≤0.8 log2 ratio, spanning from 5Mb to 40Mb of sequence and upwards of 100 genes; Figure 3b). Here the trend holds, cancer gene amplifications at relatively lower log2 ratios were associated with broad amplicons comprising many genes, and thus are less likely indicative of a driver lesion that would predict response to targeted treatment [35].

The fourth and final matched tumor set showed no cancer gene aberrations in the primary specimen according to aCGH analysis, but significant copy number aberrations were detected for a number of genes in the metastatic tumor sample; including focused, high copy number gain for *ERBB2*. FISH analysis verified aCGH *ERBB2*-negative results in the cancer-of-origin specimen, but the alternative measure that detected high ERBB2 protein-levels in the specimen by IHC leads to a conclusion that the dependence of this patient's cancer on ERBB2 was sustained in both the primary and metastatic sites by more than one mechanism. Of note, in the course of treatment for this primary breast cancer, the patient did receive anti-ERBB2 therapy as part of her treatment regimen, which a recent study has shown may actually contribute to the development of brain metastases [36]. This specific scenario challenges mutation or oncogene testing algorithms where the objective is to discover an actionable, activated oncogene in a patient specimen. While genomic approaches (e.g., aCGH, high-throughput sequencing of targeted exons or exome sequencing) conveniently allow for multi-gene testing and thus may provide benefit over single gene analysis such as by FISH, all efforts to analyze cancer specimen DNA genomic methods or by FISH can result in false negatives when an unforeseen mechanism upregulates an oncogene at the transcript or protein level. In line with the result we report here, signaling mechanisms are known to drive transcription and upregulation of ERBB2 in breast cancer absent any copy number gain [37].

To conclude, the results of this study demonstrate the potential to identify an array of known, druggable oncogenic lesions in breast metastatic brain tumor specimens – with links to the stem cell pluripotency pathway. All in all bolstering the expectation that continued molecular genomic study of primary breast cancer and metastatic brain tumors will lead to improved targeted, individualized therapies.

## MATERIALS AND METHODS

### Patients and tissues

This study was approved by the Wayne State University School of Medicine Institutional Review Board and written informed consent was obtained from all participants. Patients included in this study underwent craniotomy with microsurgical brain tumor resection as part of the standard-of-care for the treatment of breast cancer brain metastases. The study procurement period was 2009-2013. Fresh tissue specimens were collected and frozen in liquid nitrogen within 30 min post-resection and stored at −80°C until analysis. Formalin-fixed paraffin-embedded (FFPE) specimens were obtained from the Pathology archive. Patient demographic and clinical outcomes data collected included: date of birth, gender, age at diagnosis of primary breast cancer and metastatic brain tumor, and histopathological features of primary and metastatic tumors. A description of each specimen from all 10 patients who contributed to this study – including ER, ERBB2/HER2 and progesterone receptor (PR) status and treatments – is provided in Table 1.

### Array Comparative Genomic Hybridization (aCGH) analysis

The analyses were done in a CLIA-certified laboratory and CGH was run using a methodology established in that laboratory that is reviewed and accredited by the College of American Pathologists (CAP). DNA was isolated from paraffin-embedded tissue or frozen tissue using the EZ1 Advanced XL magnetic bead technology, the EZ1 DNA de-paraffinization method (where appropriate), and the EZ1 DNA Tissue kit (Qiagen, Valencia, CA). Cytogenomic analysis was done by array comparative genomic hybridization (aCGH) method. Sample DNA was pre-assessed and selected based on Nanodrop spectrophotometer outcomes to estimate quantity and contaminating factors and TapeStation (Agilent Technologies, Santa Clara, CA) digital gel technology to estimate degree of degradation, where all FFPE samples analyzed and corresponding heat-treated reference DNA were observed to be similarly fragmented (range of 1000-2000bp). aCGH was performed with Agilent SurePrint G3 ISCA CGH+SNP 180K using a normal characterized female DNA reference (Agilent Technologies). Cancer and reference DNA (500ng each) were labeled with fluorescent dyes, Cy5 and Cy3 respectively, using the SureTag Complete DNA Labeling Kit (Agilent Technologies) or Universal Linkage System labeling kit for DNA from FFPE specimens (Agilent Technologies) as in [38]. Slides were scanned using an Agilent G4900DA SureScan Microarray Scanner System, and Log2 ratios for coordinates showing copy number variation (CNV) were extracted using Agilent CytoGenomics Edition 2.5.8.1 using the ADM2 threshold set at 6; an 8 probe minimum; and an additional absolute log2 ratio threshold minimum set at 0.4 for analysis of reoccurring lesions or minimum of log2 ratio 0.8 to study high amplitude copy number aberrations. To identify genes with well-characterized roles in cancer, gene-level data were compared to a cancer gene list comprising known cancer promoting genes, oncogenes or tumor suppressor genes (Supplementary Table 1); the listed genes were compiled from the Cancer Gene Census (a cancer atlas project supported by the Wellcome Trust Sanger Institute, London, UK), and work by Lawrence *et al*. and Vogelstein *et al.* [39, 40]. Ingenuity Systems software and databases (Qiagen Silicon Valley, Redwood City, CA) were used for Pathways Enrichment Analysis and to highlight potentially druggable pathways and genes. Here the *p*-value associated with a pathway annotation is a measure of its statistical significance with respect to genes in the canonical pathway, eligible genes equal to the analyzed dataset of interest and a reference set equal to all human genes probed in the microarray. The *p*-value was calculated with the right-tailed Fisher's Exact Test. CGH data, raw and processed, are available at http://www.ncbi.nlm.nih.gov/geo/query/acc.cgi?token=ozkdeuiwjvkbbij&acc=GSE62009.

### Immunohistochemistry (IHC) and Fluorescent *In Situ* Hybridization (FISH) assays

IHC was performed using standard protocols on 5 mM FFPE tissue sections after citrate buffer antigen retrieval. The ERBB2/HER2 antibody (rabbit clone 29D8, Cell Signaling Technology, Danvers, MA) was diluted 1:75 and incubated overnight at 4°C. Antibody binding was visualized with 3,3′-diaminobenzidine and sections were counterstained with Mayer's hematoxylin before permanent mounting.

FISH was performed on FFPE tissue using the Vysis PathVysion ERBB2/HER2 DNA probe kit (Abbott Molecular Inc., Abbott Park, IL) following the manufacturer's protocol. The LSI ERBB2/HER2 DNA probe (labeled with SpectrumOrange) is specific for the ERBB2/HER2 gene locus on 17q11.2-q12 while the CEP 17 DNA control probe (labeled with SpectrumGreen is specific for the centromeric region of chromosome 17 (17q11.1-q11.1). At least 20 non-overlapping cells in two separate areas of invasive cancer were scored following the American Society of Clinical Oncology (ASCO) and the College of American Pathologists (CAP) guidelines [41].

## SUPPLEMENTARY TABLES


