# Supplementary material for: Cytogenomic profiling of breast cancer brain metastases reveals potential for repurposing targeted therapeutics
Source: Oncotarget. 2015 Apr 24;6(16):14614–24. doi: 10.18632/oncotarget.3786 (PMC4546491; doi:10.18632/oncotarget.3786)
Supplement: Supplementary file 1 [file oncotarget-06-14614-s001.pdf]

SUPPLEMENTARY TABLES

Supplementary Table S1. Cancer gene list (560 genes)

ABI1	CBL	ELK4	GRLF1	MALT1	NSD1	RET	TET1
ABL1	CBLB	ELL	H3F3A	MAML2	NT5C2	RHEB	TET2
ABL2	CBLC	ELN	H3F3B	MAP2K1	NTRK1	RHOA	TFE3
ACSL3	CCDC6	EML4	HERPUD1	MAP2K2	NTRK3	RNF213	TFEB
AFF4	CCNB1IP1	EP300	HEY1	MAP2K4	NUMA1	RNF43	TFG
AKAP9	CCND1	EPS15	HIP1	MAP4K3	NUP214	ROS1	TFPT
AKT1	CCND2	ERBB2	HIST1H3B	MAX	NUP98	RPL10	TFRC
AKT2	CCND3	ERC1	HIST1H4E	MBD1	OLIG2	RPL22	THRAP3
ALDH2	CCNE1	ERCC2	HIST1H4I	MDM2	OMD	RPL5	TIF1
ALK	CD1D	ERCC3	HLA-B	MDM4	P2RY8	RPL5	TLX1
ALPK2	CD274	ERCC4	HLF	MDS1	PAFAH1B2	RPN1	TLX3
AMER1	CD74	ERCC5	HLXB9	MDS2	PALB2	RUNDC2A	TMPRSS2
APC	CD79A	ERG	HMGA1	MECT1	PAX3	RUNX1	TNF
AR	CD79B	ETV1	HMGA2	MED12	PAX5	RUNXBP2	TNFAIP3
ARHGAP26	CDC73	ETV4	HNRNPA2B1	MEN1	PAX7	RXRA	TNFRSF14
ARHGEF12	CDH1	ETV5	HOOK3	MET	PAX8	SBDS	TNFRSF17
ARHH	CDH11	ETV6	HOXA11	MGA	PBRM1	SDC4	TNFRSF6
ARID1A	CDK12	EVI1	HOXA13	MITF	PBX1	SDHAF2	TOP1
ARID2	CDK4	EWSR1	HOXA9	MKL1	PCBP1	SDHB	TP53
ARNT	CDK6	EXT1	HOXC11	MLF1	PCM1	SDHC	TP53BP1
ASPSCR1	CDKN2A	EXT2	HOXC13	MLH1	PCSK7	SDHD	TPM3
ASXL1	CDKN2AIP	EZH1	HOXD11	MLL	PDCD1LG2	SEPT6	TPM4
ATF1	CDKN2C	EZH2	HOXD13	MLL2	PDE4DIP	SET	TPR
ATIC	CDX2	EZR	HRAS	MLL3	PDGFB	SETBP1	TPX2
ATM	CEBPA	FACL6	HSPCA	MLLT1	PDGFRA	SETD2	TRA
ATP1A1	CEP1	FAM22A	HSPCB	MLLT10	PDGFRB	SETDB1	TRAF7
ATP2B3	CEP76	FAM22B	IDH1	MLLT11	PER1	SF3B1	TRB
ATRX	CHCHD7	FAM46C	IDH2	MLLT2	PHF6	SFPQ	TRD
AURKA	CHD8	FANCA	IGH	MLLT3	PHOX2B	SFRS3	TRIM23
AXIN1	CHEK2	FANCC	IGK	MLLT4	PICALM	SH2B3	TRIM27
BAP1	CHIC2	FANCD2	IGL	MLLT6	PIK3CA	SH3GL1	TRIM33
BCL10	CHN1	FANCE	IKZF1	MLLT7	PIK3R1	SLC34A2	TRIP11
BCL11A	CIC	FANCF	IL2	MN1	PIM1	SLC45A3	TRRAP
BCL11B	CIITA	FANCG	IL21R	MPL	PLA2G10	SMARCA4	TSC1

(*Continued*)

BCL2	CLP1	FBXO11	IL6ST	MSF	PLAG1	SMARCB1	TSC2
BCL3	CLTC	FBXW7	IL7R	MSH2	PML	SMARCE1	TSHR
BCL5	CLTCL1	FCGR2B	IRF4	MSH6	PMS1	SMO	TTL
BCL6	CMKOR1	FCRL4	IRF6	MSI2	PMS2	SOCS1	U2AF1
BCL7A	CNOT3	FEV	ITK	MSN	PMX1	SOS1	UBR5
BCL9	COL1A1	FGFR1	JAK1	MTCP1	PNUTL1	SOX2	USP6
BCLAF1	COPEB	FGFR1OP	JAK2	MUC1	POT1	SPECC1	VHL
BCOR	COX6C	FGFR2	JAK3	MUTYH	POU2AF1	SRGAP3	VTI1A
BCR	CREB1	FGFR3	JAZF1	MYB	POU5F1	SRSF2	WAS
BIRC3	CREB3L1	FH	JUN	MYC	PPARG	SS18	WHSC1
BLM	CREB3L2	FHIT	KCNJ5	MYCL1	PPP2R1A	SS18L1	WHSC1L1
BMPR1A	CREBBP	FIP1L1	KDM5A	MYCN	PRCC	SSX1	WIF1
BRAF	CRLF2	FLCN	KDM5C	MYD88	PRDM1	SSX2	WRN
BRCA1	CRTC3	FLI1	KDM6A	MYH11	PRDM16	SSX4	WT1
BRCA2	CSF3R	FLT3	KDR	MYH9	PRF1	STAG2	WWTR1
BRD3	CTNNB1	FNBP1	KIAA1549	MYOCD	PRKAR1A	STAT3	XPA
BRD4	CYLD	FOXA1	KIF5B	MYST4	PSIP2	STAT5B	XPC
BRIP1	D10S170	FOXL2	KIT	NACA	PTCH	STIL	XPO1
BTG1	DAXX	FOXO1A	KLF4	NBS1	PTCH1	STK11	YWHAE
BUB1B	DDB2	FOXO3A	KLK2	NCKIPSD	PTEN	STL	ZNF145
C12orf9	DDIT3	FOXP1	KRAS	NCOA1	PTPN11	STX2	ZNF198
C15orf21	DDR2	FSTL3	KTN1	NCOA2	PTPRC	SUFU	ZNF278
C15orf55	DDX10	FUBP1	LAF4	NCOA4	QKI	SUZ12	ZNF331
C15orf65	DDX5	FUS	LASP1	NDRG1	RABEP1	SYK	ZNF384
C16orf75	DDX6	FVT1	LCK	NF1	RAC1	TAF15	ZNF521
C2orf44	DEK	GAS7	LCP1	NF2	RAD21	TAL1	ZNF750
CACNA1D	DICER1	GATA1	LHFP	NFE2L2	RAD21	TAL2	ZNF9
CALR	DNER	GATA2	LIFR	NFIB	RAD51L1	TAP1	ZRANB3
CAMTA1	DNM2	GATA3	LMO1	NFKB2	RAF1	TBL1XR1	ZRSR2
CANT1	DNMT3A	GMPS	LMO2	NIN	RALGDS	TCEA1	
CARD11	DUX4	GNA11	LPP	NKX2-1	RANBP17	TCF1	
CARS	EBF1	GNAQ	LRIG3	NONO	RAP1GDS1	TCF12	
CASC5	ECT2L	GNAS	LYL1	NOTCH1	RARA	TCF3	
CASP8	EGFR	GOLGA5	MADH4	NOTCH2	RB1	TCF7L2	
CBFA2T1	EIF4A2	GOPC	MAF	NPM1	RBM15	TCL1A	
CBFA2T3	ELF3	GPC3	MAFB	NR4A3	RECQL4	TCL6	
CBFB	ELF4	GPHN	MALAT1	NRAS	REL	TERT	

Supplementary Table S2. 55 cancer genes showed copy number variation (CNV) ≥0.4 log2 ratio in ≥40% of the breast metastatic brain tumors analyzed (*n* = 10)

Gene Symbol	Mean Amplitude (log2 ratio)	Minimum Amplitude (log2 ratio)	Maximum Amplitude (log2 ratio)	Number of Specimens with CNV
TERT	1.01	0.55	1.75	4
ARNT	0.98	0.75	1.45	4
MLLT11	0.98	0.75	1.45	4
SETDB1	0.98	0.75	1.45	4
NCOA2	0.92	0.41	2.03	4
HEY1	0.88	0.41	2.21	5
FH	0.86	0.66	1.39	4
TPM3	0.82	0.75	0.92	4
MUC1	0.82	0.75	0.92	4
BCL9	0.81	0.75	0.88	4
H3F3A	0.81	0.43	1.55	5
IL7R	0.80	0.55	1.22	4
LIFR	0.80	0.55	1.22	4
PIK3CA	0.78	0.52	1.05	4
CD1D	0.78	0.75	0.85	4
PDE4DIP	0.78	0.75	0.85	4
PRCC	0.78	0.75	0.85	4
FCGR2B	0.78	0.75	0.85	4
FCRL4	0.78	0.75	0.85	4
SDHC	0.78	0.75	0.85	4
NTRK1	0.78	0.75	0.85	4
ETV5	0.77	0.52	1.02	4
BCL6	0.77	0.52	1.02	4
LPP	0.77	0.52	1.02	4
EIF4A2	0.77	0.52	1.02	4
HOOK3	0.75	0.44	1.04	4
TBL1XR1	0.74	0.52	1.13	4
WWTR1	0.73	0.46	1.03	5
IRF4	0.72	0.54	0.93	4
DEK	0.72	0.54	0.93	4
EXT1	0.71	0.44	0.91	6
TRIM27	0.70	0.54	1.11	4
HIST1H4I	0.70	0.54	1.11	4
RAD21	0.70	0.41	0.91	6

(*Continued*)

Gene Symbol	Mean Amplitude (log2 ratio)	Minimum Amplitude (log2 ratio)	Maximum Amplitude (log2 ratio)	Number of Specimens with CNV
MYC	0.70	0.44	0.91	6
SOX2	0.67	0.52	0.84	4
HIST1H3B	0.66	0.54	0.93	4
HIST1H4E	0.66	0.54	0.93	4
GMPS	0.66	0.46	0.91	4
NDRG1	0.65	0.41	0.91	5
UBR5	0.65	0.41	0.91	7
CHCHD7	0.64	0.41	0.91	4
PLAG1	0.64	0.41	0.91	4
TCEA1	0.64	0.41	0.91	4
COX6C	0.63	0.41	0.91	7
RECQL4	0.62	0.41	0.91	5
CARD11	0.58	0.45	0.75	4
ELN	0.57	0.44	0.71	5
HIP1	0.57	0.44	0.71	5
SBDS	0.56	0.50	0.66	4
SS18L1	0.56	0.46	0.85	5
HOXD13	0.55	0.45	0.64	4
HOXD11	0.55	0.45	0.64	4
RPL5	0.48	0.40	0.56	4
COL1A1	0.46	0.40	0.51	4

Supplementary Table 3. 109 cancer genes showed copy number variation (CNV) ≥ 0.8 log2 ratio in at least 1 of the breast metastatic brain tumors analyzed (*n* = 10)

Gene Symbol	Mean Amplitude (log2 ratio)	Minimum Amplitude (log2 ratio)	Maximum Amplitude (log2 ratio)	Number of Specimens with CNV	Function	Inhibitor Drug (e.g.)
CDK12	2.46	1.83	3.10	2	kinase	
MAML2	2.35	2.35	2.35	1	transcription regulator	
HEY1	2.21	2.21	2.21	1	transcription regulator	
ERBB2	2.19	1.83	2.54	2	kinase	yes (trastuzumab, lapatinib, erlotinib)
LASP1	2.19	1.83	2.54	2	transporter	
CD274	2.17	2.17	2.17	1	enzyme	
JAK2	2.17	2.17	2.17	1	kinase	yes (ruxolitinib)
PDCD1LG2	2.17	2.17	2.17	1	enzyme	
CCND1	2.11	2.11	2.11	1	other	yes (daunorubicin, gemtuzumab)
MLLT6	2.07	1.59	2.54	2	transcription regulator	
NCOA2	2.03	2.03	2.03	1	transcription regulator	
PICALM	1.77	1.77	1.77	1	other	
FGFR1	1.75	1.75	1.75	1	kinase	yes (sorafenib, dexamethasone)
WHSC1L1	1.75	1.75	1.75	1	enzyme	
CHIC2	1.57	0.88	2.26	2	other	
PDGFRA	1.57	0.88	2.26	2	kinase	yes (sunitinib, pazopanib, imatinib)
H3F3A	1.55	1.55	1.55	1	other	
TERT	1.48	1.21	1.75	2	enzyme	
MYB	1.44	0.81	2.59	3	transcription regulator	
SUZ12	1.43	1.43	1.43	1	enzyme	
TAF15	1.43	1.43	1.43	1	other	
ZNF331	1.40	1.40	1.40	1	other	
FH	1.39	1.39	1.39	1	enzyme	
FIP1L1	1.39	0.88	1.89	2	other	
HLF	1.35	1.35	1.35	1	transcription regulator	
MYCN	1.26	1.26	1.26	1	transcription regulator	

(*Continued*)

Gene Symbol	Mean Amplitude (log2 ratio)	Minimum Amplitude (log2 ratio)	Maximum Amplitude (log2 ratio)	Number of Specimens with CNV	Function	Inhibitor Drug (e.g.)
TBL1XR1	1.13	1.13	1.13	1	transcription regulator	
CBLB	1.12	1.12	1.12	1	other	
TFG	1.12	1.12	1.12	1	other	
CNOT3	1.12	1.12	1.12	1	other	
TFPT	1.12	1.12	1.12	1	other	
HIST1H4I	1.11	1.11	1.11	1	other	
TRIM27	1.11	1.11	1.11	1	transcription regulator	
TFRC	1.07	1.07	1.07	1	transporter	
ARNT	1.06	0.85	1.45	3	transcription regulator	
MLLT11	1.06	0.85	1.45	3	other	
SETDB1	1.06	0.85	1.45	3	enzyme	
ZRSR2	1.05	1.05	1.05	1	other	
IL7R	1.04	0.86	1.22	2	transmembrane receptor	
LIFR	1.04	0.86	1.22	2	transmembrane receptor	
HOOK3	1.03	1.01	1.04	2	other	
BLM	1.02	1.02	1.02	1	enzyme	
CRTC3	1.02	1.02	1.02	1	other	
IDH2	1.02	1.02	1.02	1	enzyme	
NTRK3	1.02	1.02	1.02	1	kinase	
MLLT10	1.02	1.02	1.02	1	transcription regulator	
WRN	1.01	1.01	1.01	1	enzyme	
PBX1	0.99	0.99	0.99	1	transcription regulator	
TPR	0.99	0.99	0.99	1	transporter	
CTNNB1	0.99	0.99	0.99	1	transcription regulator	
MYD88	0.99	0.99	0.99	1	other	
PPP2R1A	0.98	0.84	1.12	2	phosphatase	
WWTR1	0.97	0.91	1.03	2	transcription regulator	
CCNE1	0.96	0.80	1.13	2	transcription regulator	

(*Continued*)

Gene Symbol	Mean Amplitude (log2 ratio)	Minimum Amplitude (log2 ratio)	Maximum Amplitude (log2 ratio)	Number of Specimens with CNV	Function	Inhibitor Drug (e.g.)
PIK3CA	0.94	0.84	1.05	2	kinase	yes (SF 1126, PX-866)
HIST1H3B	0.93	0.93	0.93	1	other	
HIST1H4E	0.93	0.93	0.93	1	other	
BCL6	0.93	0.84	1.02	2	transcription regulator	
EIF4A2	0.93	0.84	1.02	2	translation regulator	
ETV5	0.93	0.84	1.02	2	transcription regulator	
LPP	0.93	0.84	1.02	2	other	
SOS1	0.92	0.92	0.92	1	other	
GOPC	0.91	0.91	0.91	1	transporter	
ROS1	0.91	0.91	0.91	1	kinase	
TPX2	0.91	0.91	0.91	1	other	
FOXL2	0.91	0.91	0.91	1	transcription regulator	
GMPS	0.91	0.91	0.91	1	enzyme	
MLF1	0.91	0.91	0.91	1	other	
CHCHD7	0.91	0.91	0.91	1	other	
PLAG1	0.91	0.91	0.91	1	transcription regulator	
RECQL4	0.91	0.91	0.91	1	enzyme	
TCEA1	0.91	0.91	0.91	1	transcription regulator	
DEK	0.90	0.87	0.93	2	transcription regulator	
IRF4	0.90	0.87	0.93	2	transcription regulator	
COX6C	0.89	0.87	0.91	2	enzyme	
EXT1	0.89	0.87	0.91	2	enzyme	
MYC	0.89	0.87	0.91	2	transcription regulator	
RAD21	0.89	0.87	0.91	2	other	
UBR5	0.89	0.87	0.91	2	enzyme	
MUC1	0.89	0.85	0.92	2	transcription regulator	yes (HuHMFG1)

(*Continued*)

Gene Symbol	Mean Amplitude (log2 ratio)	Minimum Amplitude (log2 ratio)	Maximum Amplitude (log2 ratio)	Number of Specimens with CNV	Function	Inhibitor Drug (e.g.)
TPM3	0.89	0.85	0.92	2	other	
KDR	0.88	0.88	0.88	1	kinase	yes (cabozantinib, bevacizumab)
KIT	0.87	0.86	0.88	2	transmembrane receptor	yes (dasatinib, sunitinib)
BCL9	0.87	0.85	0.88	2	other	
NDRG1	0.87	0.82	0.91	2	kinase	
RAF1	0.86	0.86	0.86	1	kinase	yes (vemurafenib, sorafenib)
BCLAF1	0.86	0.81	0.91	2	transcription regulator	
STL	0.86	0.81	0.91	2	other	
ABL2	0.85	0.85	0.85	1	kinase	
CD1D	0.85	0.85	0.85	1	other	
DDR2	0.85	0.85	0.85	1	kinase	yes (regorafenib)
FCGR2B	0.85	0.85	0.85	1	transmembrane receptor	
FCRL4	0.85	0.85	0.85	1	other	
NTRK1	0.85	0.85	0.85	1	kinase	yes (regorafenib)
PDE4DIP	0.85	0.85	0.85	1	enzyme	
PRCC	0.85	0.85	0.85	1	other	
SDHC	0.85	0.85	0.85	1	enzyme	
GNAS	0.85	0.85	0.85	1	enzyme	
SS18L1	0.85	0.85	0.85	1	transcription regulator	
GATA2	0.84	0.84	0.84	1	transcription regulator	
RPN1	0.84	0.84	0.84	1	enzyme	
SOX2	0.84	0.84	0.84	1	transcription regulator	
CLP1	0.83	0.83	0.83	1	other	
SDHAF2	0.83	0.83	0.83	1	other	
ECT2L	0.81	0.81	0.81	1	other	
BRD4	0.81	0.81	0.81	1	kinase	
ELL	0.81	0.81	0.81	1	transcription regulator	
JAK3	0.81	0.81	0.81	1	kinase	yes (tofacitinib, R-348)
TPM4	0.81	0.81	0.81	1	other	
